# Supplementary material for: Yiai Fuzheng decoction inhibits triple-negative breast cancer by remodeling the immune microenvironment
Source: Front Immunol. 2025 Sep 30;16:1615631. doi: 10.3389/fimmu.2025.1615631 (PMC12518410; doi:10.3389/fimmu.2025.1615631)
Supplement: Supplementary file 2 [file DataSheet1.zip › Supplementary File 2/Treatment/C_vs_H/07_Pathway_Analysis/Pathway_Analysis/Sig_Pathways_ID/Arachidonic acid metabolism.pdf]

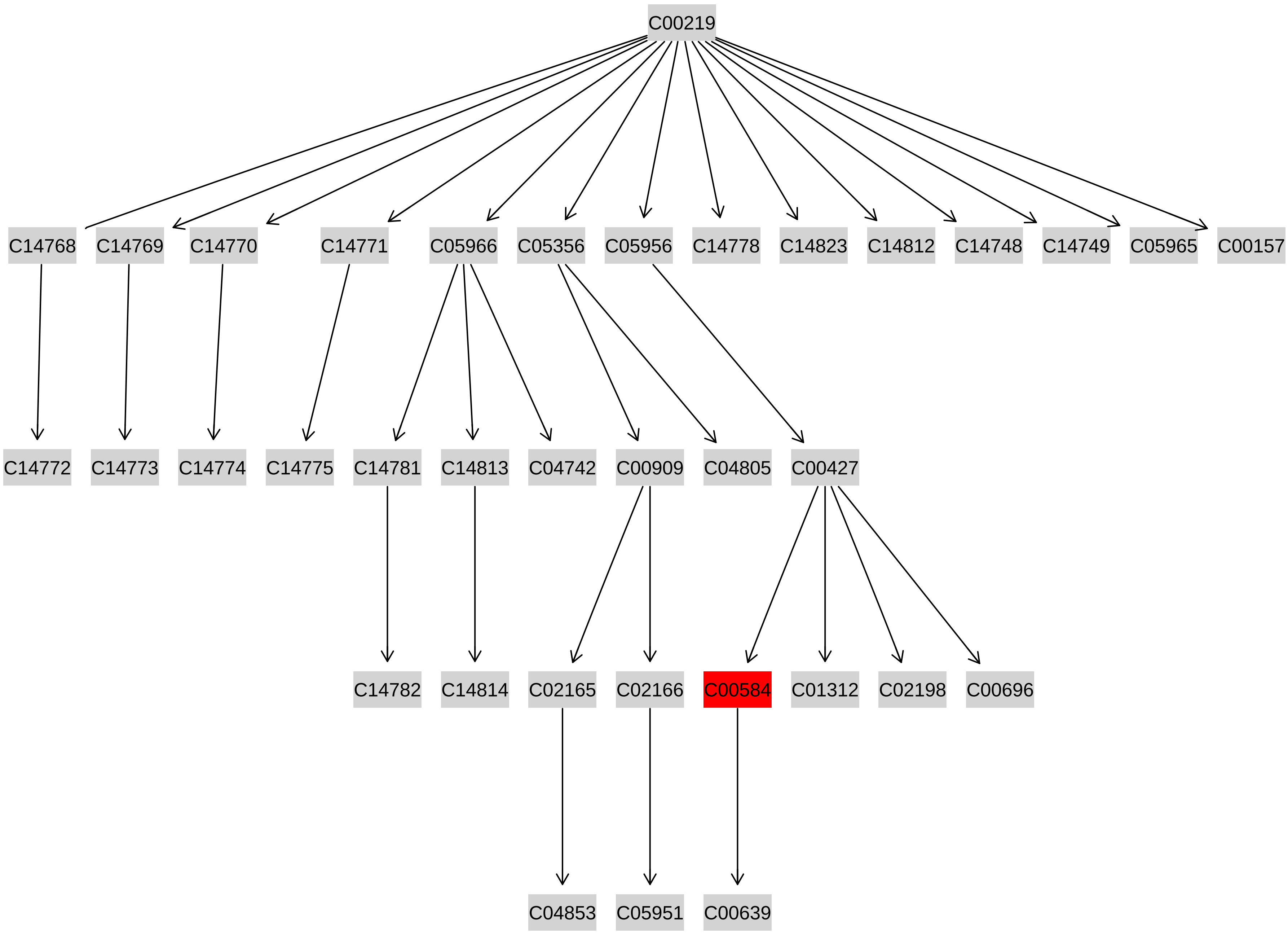
C00219
C14768
C14769
C14770
C14771
C05966
C05356
C05956
C14778
C14823
C14812
C14748
C14749
C05965
C00157
C14772
C14773
C14774
C14775
C14781
C14813
C04742
C00909
C04805
C00427
C14782
C14814
C02165
C02166
C00584
C01312
C02198
C00696
C04853
C05951
C00639
